# FRET-SLiM on native autofluorescence: a fast and reliable method to study interactions between fluorescent probes and lignin in plant cell wall

**DOI:** 10.1186/s13007-018-0342-3

**Published:** 2018-08-27

**Authors:** Christine Terryn, Gabriel Paës, Corentin Spriet

**Affiliations:** 1 PICT Platform, University of Reims Champagne-Ardenne, 51 rue Cognacq-Jay, 51100 Reims, France; 2 Fractionation of AgroResources and Environment (FARE) Laboratory, INRA, University of Reims Champagne-Ardenne, 2 Esplanade Roland-Garros, 51100 Reims, France; 3 TISBio, Unité de Glycobiologie Structurale et Fonctionnelle (UGSF), CNRS, UMR 8576, Université de Lille, 59000 Lille, France

**Keywords:** SLiM, Fluorescence lifetime, FRET, Autofluorescence, Lignocellulose, Interaction

## Abstract

**Background:**

Lignocellulosic biomass is a complex network of polymers making the cell walls of plants. It represents a feedstock of sustainable resources to be converted into fuels, chemicals and materials. Because of its complex architecture, lignocellulose is a recalcitrant material that necessitates some pretreatments and several types of catalysts to be transformed efficiently. In particular, enzymes degrading lignocellulose can become inactivated due to their binding to lignin through non-specific interactions, leading to a loss in catalytic efficiency of industrial processes. Gaining more knowledge in the strength of interactions would allow optimizing enzymes and selecting appropriate pretreatments.

**Results:**

Measuring interactions directly in plant cell wall can theoretically be performed using confocal fluorescence techniques by evaluating fluorescence resonance energy transfer (FRET) between compatible fluorophores. In this study, autofluorescence of plant cell wall, mainly originating from lignin, was considered as a donor fluorophore while the acceptor was a common rhodamine-based fluorescent probe. To overcome complex plant cell wall fluorescence, which limits FRET analysis by standard techniques, we have developed an original approach, combining spectral and lifetime measurements. It consists in (1) dissecting autofluorescence signal in each spectral channel, (2) optimizing spectral channel choice for lifetime measurements and (3) achieving an unambiguous FRET signature with an autofluorescent donor fluorophore. Interactions between rhodamine-based probes of various sizes and untreated or pretreated wheat sample were evaluated, showing it was possible to discriminate interactions at the nano-scale, revealing some accessibility differences and the effect of pretreatment.

**Conclusions:**

SLiM measurement allows precise estimation of the optimal spectral range for FRET measurement. SLiM response allows for the first time doubtless FRET measurements between lignin as a donor, and an acceptor fluorophore with high accuracy and sensitivity related to lifetime decrease studies. As demonstrated, it thus becomes possible to measure interactions of fluorescent probes directly inside plant cell wall samples. This approach can thus be applied to various fields such as lignocellulose deconstruction to optimize the action of enzymes or plant cell wall development to assay in situ the biosynthesis of lignin.

**Electronic supplementary material:**

The online version of this article (10.1186/s13007-018-0342-3) contains supplementary material, which is available to authorized users.

## Background

Lignocellulose is the plant framework made through photosynthesis, so it is considered as the most important renewable carbon resource that could contribute to replace industrial fossil carbon dependency [1]. But lignocellulose is a very complex network of polysaccharides (mainly cellulose and hemicelluloses) and polyphenols (lignin) which are difficult to extract and to transform optimally [2]. That is why some physico-chemical pretreatments [3, 4] are often applied to open the polymer network in order to favour the accessibility and the action of green and specific catalysts such as enzymes [5, 6]. In most cases, accessibility of polysaccharides is increased, together with that of lignin, whose structure and composition can be altered [7, 8]. As a result, due to its high hydrophobicity, lignin has the capacity to stick proteins such as enzymes more or less irreversibly [9–11]. Being not available for catalysis events, enzymes become inactive, so that the cost of enzymes in hydrolysis process can be a limiting factor.

Therefore, it is critical to assay the interactions of enzymes, in order to understand their behaviour to select appropriate enzyme properties and pretreatments [12]. Usually, determination of binding properties is performed whether with chemically simple oligomers or polymers not representative of the plant cell wall architecture [13, 14] or with bulk extracted/residual lignin or pretreated lignocellulose [9, 15–17]. Recent advancements have been carried out with the use of bioinspired lignocellulose assemblies [18–20]. Nonetheless, there is still a lack of technical approaches to assay the interactions of enzymes in plant materials at the cellular scale.

One of the most common way to determine interactions at the molecular scale in cells is to measure fluorescence (or Förster) resonance energy transfer (FRET) [21, 22]. Such a transfer occurs between two fluorophores, when one of them called the donor transfers its energy to another fluorophore, called the acceptor, without photon emission [23]. This transfer requires that the donor emission spectrum is partially superimposed to the acceptor spectrum. For the FRET to happen, both fluorophores must also be in close vicinity, since FRET efficiency decreases with the sixth power of their distance. Other contingencies related to the orientations between the fluorophore dipoles have to be taken into account. FRET can be measured by different techniques: sensitized emission, in which the variation in acceptor emission fluorescence is followed; acceptor photobleaching, in which the donor emission fluorescence is measured before and after acceptor photobleaching; lifetime measurement, which decreases for the donor in the presence of the acceptor [24]. The two first methods can only give qualitative results, while lifetime measurement is more sensitive and provides more quantitative data for evaluating the interactions between fluorophores [25, 26].

FRET has already been applied to measure interaction of cellulases with cellulose [27] and within fibres [28, 29] which are extracted materials from plant cell wall. In more relevant studies, interactions of pretreated pine samples with fluorescent polyethylene-glycol (PEG) [30] and enzymes [16] were also successfully investigated by FRET. In this latter case, the acceptor photobleaching technique was used, and thus suffers from inherent limits. Indeed, to be reliable, the methods needs a nearly total extinction of the acceptors without unspecific photobleaching or photoconversion of the donor, which is far to be trivial with a complex fluorophore such as lignin. Furthermore, the technique is qualitative, presents a low sensitivity and is irreversible. It is thus restricted to qualitative and static estimation of strong interactions. FRET measurement using lignin autofluorescence as a donor remains highly challenging for more quantitative studies, for mild and transient interactions or for dynamic studies. Furthermore, fluorescence lifetime imaging microscopy (FLIM), the most precise and sensitive method for FRET measurements, cannot be directly applied to study FRET using lignin autofluorescence. Indeed, lignin is a very complex donor made of different fluorophores connected through different cross-linkages [31–33], that leads to complex autofluorescence lifetime signal. Significantly, the donor’s lifetime decrease, traditionally associated with a FRET event can also be related to differences in lignin organisation and interactions. This method alone can thus lead to biased FRET estimation.

Considering the need for measuring the interactions of fluorescent probes (in particular of enzymes) with lignin in plant cell wall using quantitative techniques, we have developed a new method, using confocal microscopy and correlating two highly complementary methods for FRET quantification: fluorescence spectral and lifetime measurement (SLiM) using lignocellulose autofluorescence as a donor. Such approach provides unique FRET signature and allows for the first time quantitative and sensitive measurements of enzymes interaction in plant material.

## Results and discussion

While fluorescence lifetime measurement is the method of choice to quantify FRET events, it can lead to biased interpretation when applied to complex samples, especially when autofluorescence cannot be neglected. We previously demonstrated that correlating fluorescence lifetime and fluorescence spectrum measurements, one can achieve FRET quantification in such samples [34, 35]. In the present study, instead of taking into account autofluorescence contribution to the overall lifetime decrease, we propose a new method using lignin autofluorescence as the donor fluorophore. This method allows (1) precise estimation of the optimal spectral range for lifetime measurement, (2) unambiguous discrimination of biophysical event inducing lignin lifetime decrease, (3) FRET measurement with high accuracy and sensitivity.

**Fig. 1:**
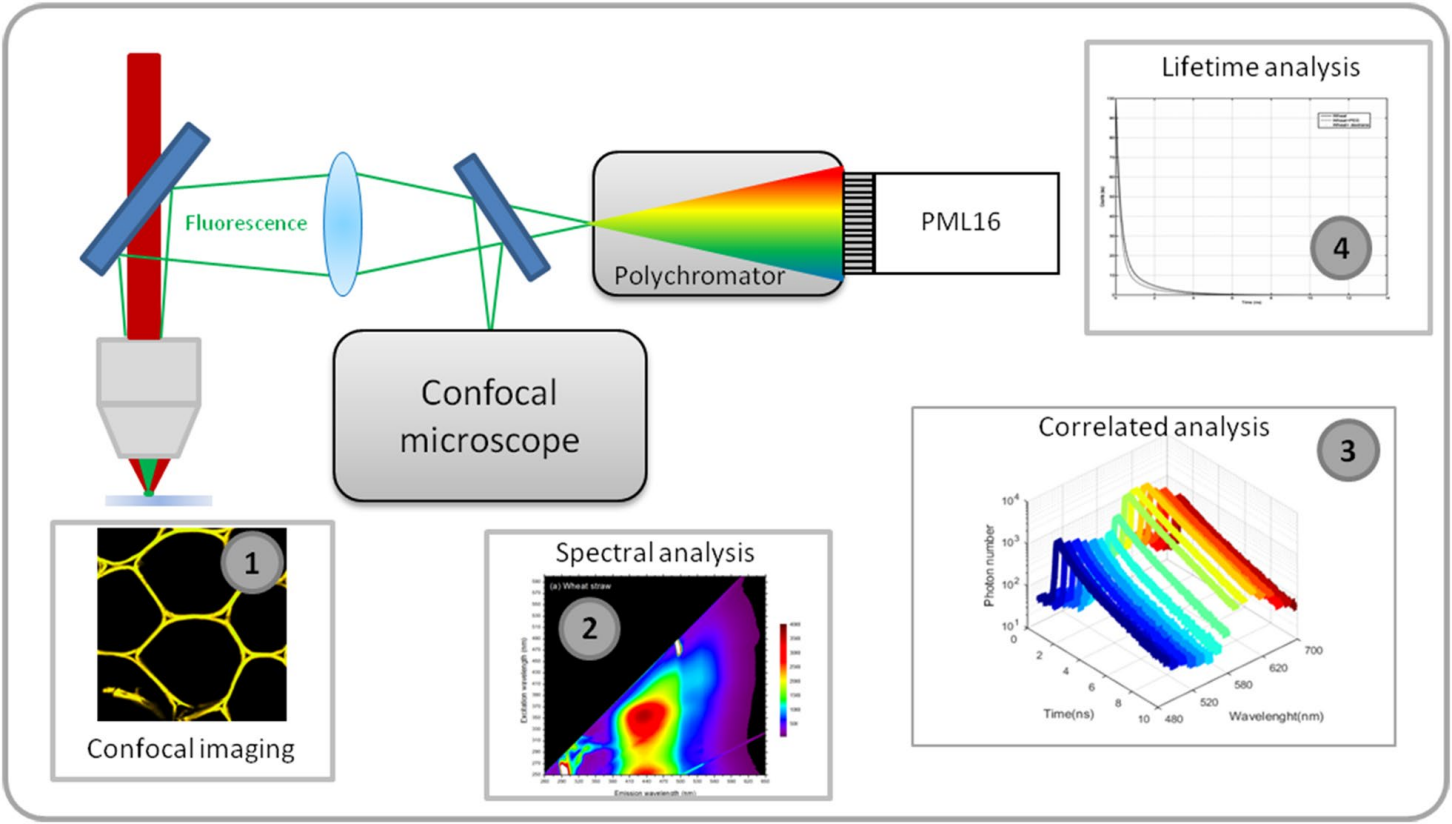
Overall process of the FRET measurement by SLiM. Confocal images of the plant cell wall sample are acquired (1) and a spectral characterisation is performed to determine optimal acquisition conditions by measuring the autofluorescence of the fluorescent probe to be assayed (2). Correlated spectral and lifetime analysis (3) then allows to determine unambiguous FRET signature while careful lifetime analysis (4) provides a quantitative FRET estimation between the fluorescent probe and the plant cell wall sample

The overall acquisition and analysis process is presented in Fig. 1. First, the plant cell wall sample is imaged in a confocal microscope on the 420–670 nm spectral range (1). After a spectral analysis of the fluorescent probes to be tested (2), the fluorescence lifetime of the plant cell wall sample is measured between 455 and 655 nm on 16 different spectral channels simultaneously, using SLiM time correlated single photon counting (TCSPC) detector (3). The autofluorescence lifetime of each channel is determined for the cell wall sample alone or in the presence of a fluorescent probe which is the acceptor (4). Thus, a spectral channel is precisely determined for which specific FRET interaction can be detected. As autofluorescence of cell wall is a highly complex signal, using this autofluorescence as a FRET donor requires to precisely know how lifetime can vary depending on spectral channels. To achieve this goal, SLiM technique was selected to determine an autofluorescence lifetime signature on each spectral channel, combined with FRET measurement on the cell wall.

### Spectral FRET

The selected plant cell wall sample was wheat straw (WS), which is a representative model monocot. WS has an important autofluorescence due mainly to the presence of phenolic compounds contained in lignin [36, 37]. Fluorescence contour map of WS measured by spectrofluorimetry showed that autofluorescence was maximal for an excitation ca. 360 nm, giving a maximum emission ca. 440 nm (Additional file 1: Figure 1a). Since emission range was very large, a compatible fluorophore for FRET likely to have an excitation spectrum overlapping the emission spectrum of WS should have an excitation maximum above 510 nm in order to avoid cross-excitation. Examination of the fluorophores available in the literature indicates that rhodamine B fluorophore has a maximum excitation ca. 540–550 nm and is easily available commercially as conjugated to different biopolymers to make fluorescent probes. Fluorescence contour map of rhodamine B clearly indicates that maximum excitation of this fluorophore occurs ca. 560 nm (Additional file 1: Figure 1b). Excitation and emission spectra of WS and rhodamine B shows that they are compatible for FRET (Additional file 1: Figure 3c): WS can be considered as the donor and rhodamine B as the acceptor.

**Fig. 2:**
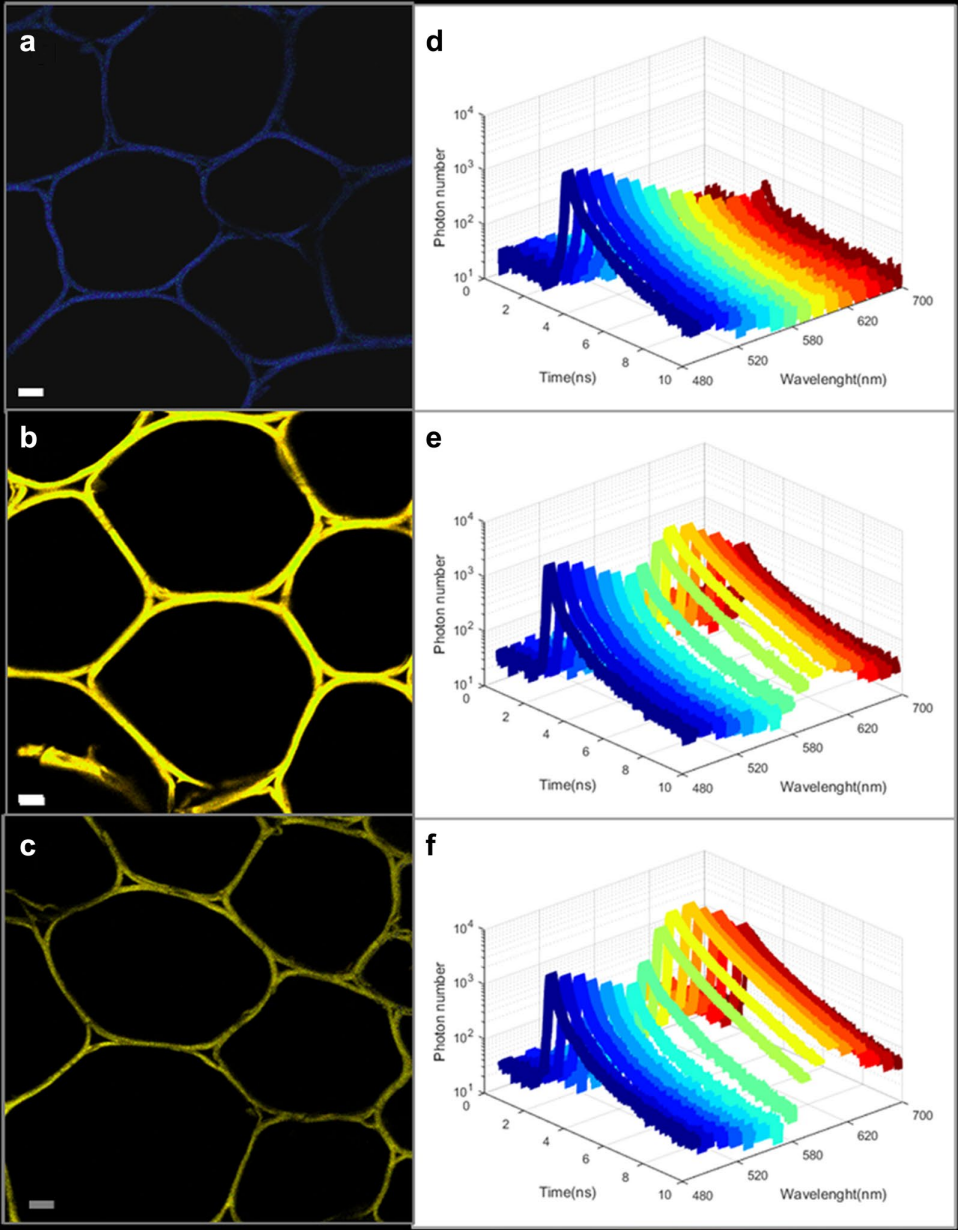
Spectral coded confocal imaging and fluorescence lifetime decay curves of **a**, **d** WS, **b**, **e** WS + PR10, **c**, **f** WS + DR10 for the 16 channels analysed

Samples were imaged in confocal microscopy, using a bi-photon excitation at 750 nm that corresponds to maximal excitation efficiency of WS. FRET measurement by spectral analysis was first performed between WS and two different types of rhodamine-based fluorescent probes: poly-ethylene-glycol-rhodamine of 10 kDa (PR10) and dextran–rhodamine of 10 kDa (DR10). Imaging fluorescence of WS, WS + PR10 and WS + DR10 showed that the fluorescence signal was in the blue region for WS (around 470 nm) (Fig. 2a), while it is more in the yellow region in the presence of a rhodamine probe (around 570 nm), with a higher fluorescence intensity for the PR10 probe (Fig. 2b) than for the DR10 probe (Fig. 2c).

Spectral analysis (Additional file 1: Figure 1c) indicated that WS presents a strong fluorescence emission peak between 440 and 550 nm while fluorescent probes based on the rhodamine fluorophore have emission ranging between 550 and 650 nm when excited at 750 nm. When WS and PR10 were incubated together, a strong decrease in WS autofluorescence emission was associated with a strong increase of rhodamine fluorescence emission with a maximum at 580 nm (Additional file 1: Figure 1c). In comparison, WS and DR10 incubation resulted in a lower decrease of the WS autofluorescence emission while the increase of rhodamine fluorescence emission was as important as for PR10, with a maximum emission shifted at 575 nm (Additional file 1: Figure 1c). Consequently, this spectral analysis showed that the fluorescence lifetime measurements must be performed between 460 and 490 nm to ensure maximum plant cell wall autofluorescence detection with no rhodamine bleed-through.

Overall, both imaging and spectral analysis of WS with the two rhodamine-based probes have shown that a FRET event occurred in both cases, but PR10 has induced a stronger quenching of WS autofluorescence than DR10, revealing that the PEG molecules seem to interact more strongly than the dextran molecules.

### SLiM measurements

With the goal of carrying out a quantitative analysis of the FRET between WS and the rhodamine probes, a SLiM acquisition system was setup (Fig. 1), allowing lifetime acquisition on 16 simultaneous channels ranging from 455 to 655 nm, thus providing correlated spectral and lifetime measurements. For WS, the fluorescence lifetime decay obtained was multiexponential as previously demonstrated [37, 38]. In order to fit this decay curve, exponential models with 1–3 parameters were tested. The 2-exponential model presented an optimal fit according to both χ^2^ and curve fit residuals, so this model was selected (Eq. 1).

**Fig. 3:**
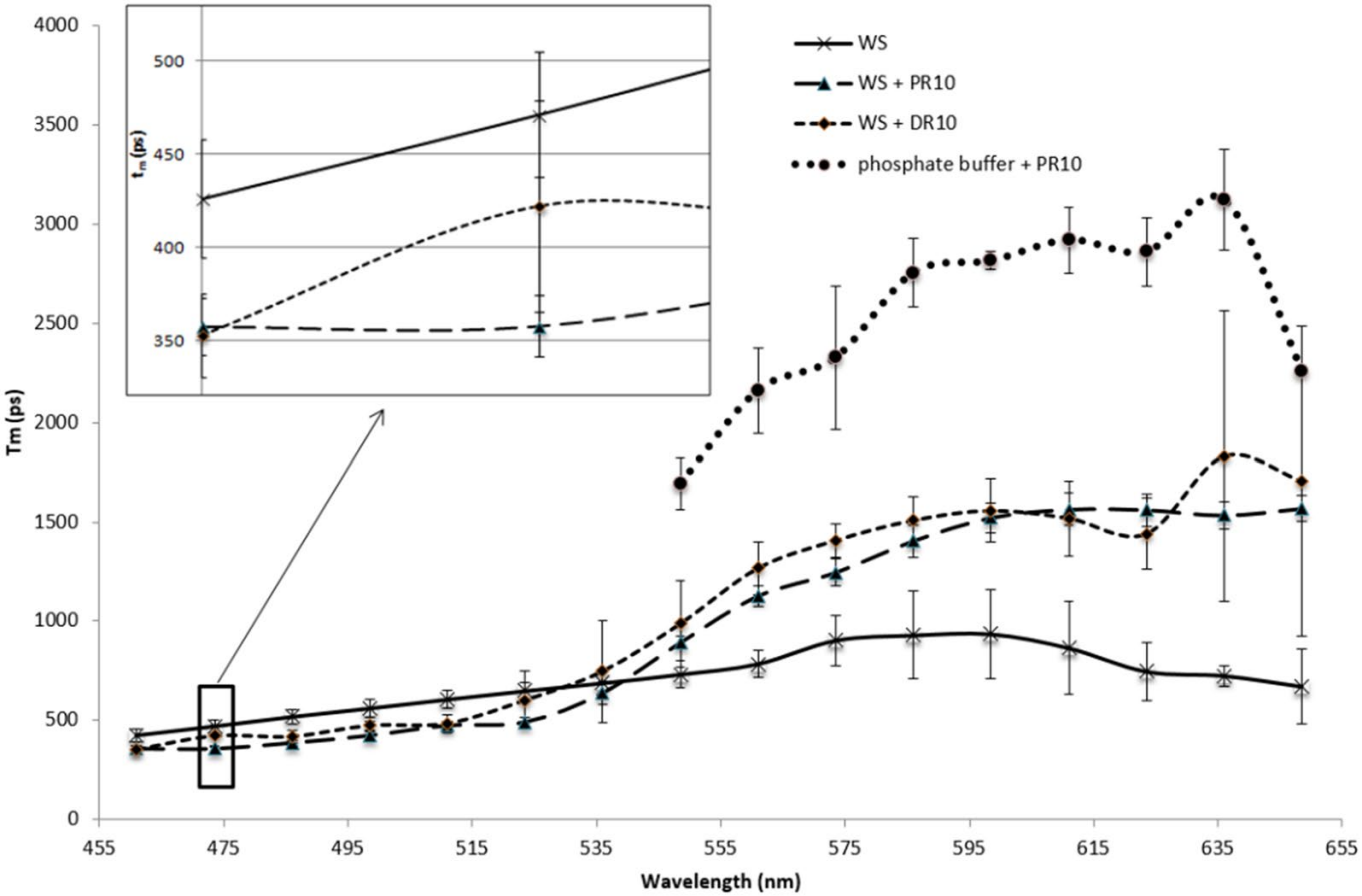
Evolution of *T*_m_ according to wavelength channels analysed for WS, WS + PR10, WS + DR10 and phosphate buffer + PR10. Inset indicates the *T*_m_ for channel 2 which is selected for FRET evaluation. Phosphate buffer + PR10 sample did not have any component in channel 2

Different samples were analysed: WS alone, WS + PR10, WS + DR10 and phosphate buffer + PR10. For each of them, the parameters related to the 2-exponential model of the fluorescence lifetime decay (*a*_1_, *a*_2_, *t*_1_, *t*_2_) were calculated, for each channel (Additional file 2: Figure 1). For facilitating comparison between the samples, the mean lifetime *T*_m_ was calculated (according to Eq. 2) and the evolution of *T*_m_ according to channel was determined (Fig. 3). For WS alone, *T*_m_ values raised from 426 ps for channel 1 (455–468 nm) to 671 ps for channel 16 (643–655 nm). A different behaviour was observed for WS incubated with PR10 and DR10. For WS + PR10, *T*_m_ values began at a lower value of 358 ps for channel 1 (455–468 nm) to reach 1567 ps for channel 16 (643–655 nm). For WS + DR10, *T*_m_ values were also spanning on a similar range from 352 ps for channel 1 (455–468 nm) to 1706 ps for channel 16 (643–655 nm).

While fluorescence lifetime is the most sensitive and accurate method to quantify FRET, it can suffer from artefacts, in particular when using complex donor fluorophores such as lignin. For example, it was demonstrated that lignin autofluorescence was affected by the compression state of wood [31]. Thus, a lifetime decrease compared to reference sample can be interpreted either by interaction with the acceptor or by a change in molecular architecture. However, in this previous study, Donaldson et al. measured fluorescence lifetime counting all photons issued from the sample without any spectral selection and thus, without specificity about autofluorescence component. They then obtained a global lifetime decrease associated to wood compaction. The method thus cannot be applied to FRET studies. With our new method, fluorescence lifetime evolution along the fluorescence spectrum can be reached and provides more insight in lignin behaviour. Notably upon addition of PR10 and DR10 to WS, we observed an homogeneous decrease of lifetime in channels between 450 to 550 nm corresponding to autofluorescence of lignin correlated to a strong lifetime increase due to acceptor sensitized emission. Indeed, the acceptor fluorescence lifetime is much higher (~ 2 ns) than that of lignin (~ 0.5 ns) and even a slight sensitized emission will result in such a modification in the SLiM mean lifetime profile.

According to spectral analysis (Additional file 1: Figure 1), WS fluorescence emission was maximum at 475 nm after a bi-photon excitation at 750 nm while emission of rhodamine was negligible, avoiding spectral overlap between the fluorescence emission of WS and rhodamine. Since the 475 nm wavelength is encompassed in the range 467.5–480 nm (Additional file 2: Figure 1), channel 2 was selected for lifetime analysis (Fig. 3 and Additional file 3: Figure 1). First, comparison of photon decay curves indicates that the slope of the WS + PR10 sample was much more pronounced than those of WS and WS + DR10 which are very close to each other, suggesting a lower fluorescence lifetime (Additional file 3: Figure 1). More precisely, calculated mean fluorescence lifetime (Table 1) of WS was 471 ps when considered alone, but was largely decreased by 24% (to 358 ps) when in the presence of PR10 and only slightly diminished by 10% (to 422 ps) with DR10. Analysis of the parameters (Table 1) showed that this discrepancy mainly originates from a decrease of both *t*_1_ and *t*_2_ values, while *a*_1_ and *a*_2_ coefficients were unchanged. The corresponding FRET efficiency (*E*_FRET_) calculated from Eq. 3 was thus higher (24%) for WS + PR10 than for WS + DR10 (10%) (Table 1).

**Table 1:** SLiM detailed parameters of channel 2 and FRET efficiency for WS, WS + PR10 and WS + DR10

	*a*_1_	*t*_1_	*a*_2_	*t*_2_	*T*_m_ (ps)	E_FRET_ (%)
WS	82 ± 1	249 ± 12	18 ± 1	1484 ± 68	471 ± 34	–
WS + PR10	85 ± 1	207 ± 12	15 ± 1	1207 ± 66	358 ± 16	24 ± 3
WS + DR10	86 ± 1	247 ± 12	15 ± 1	1446 ± 66	422 ± 16	10 ± 1

**Fig. 4:**
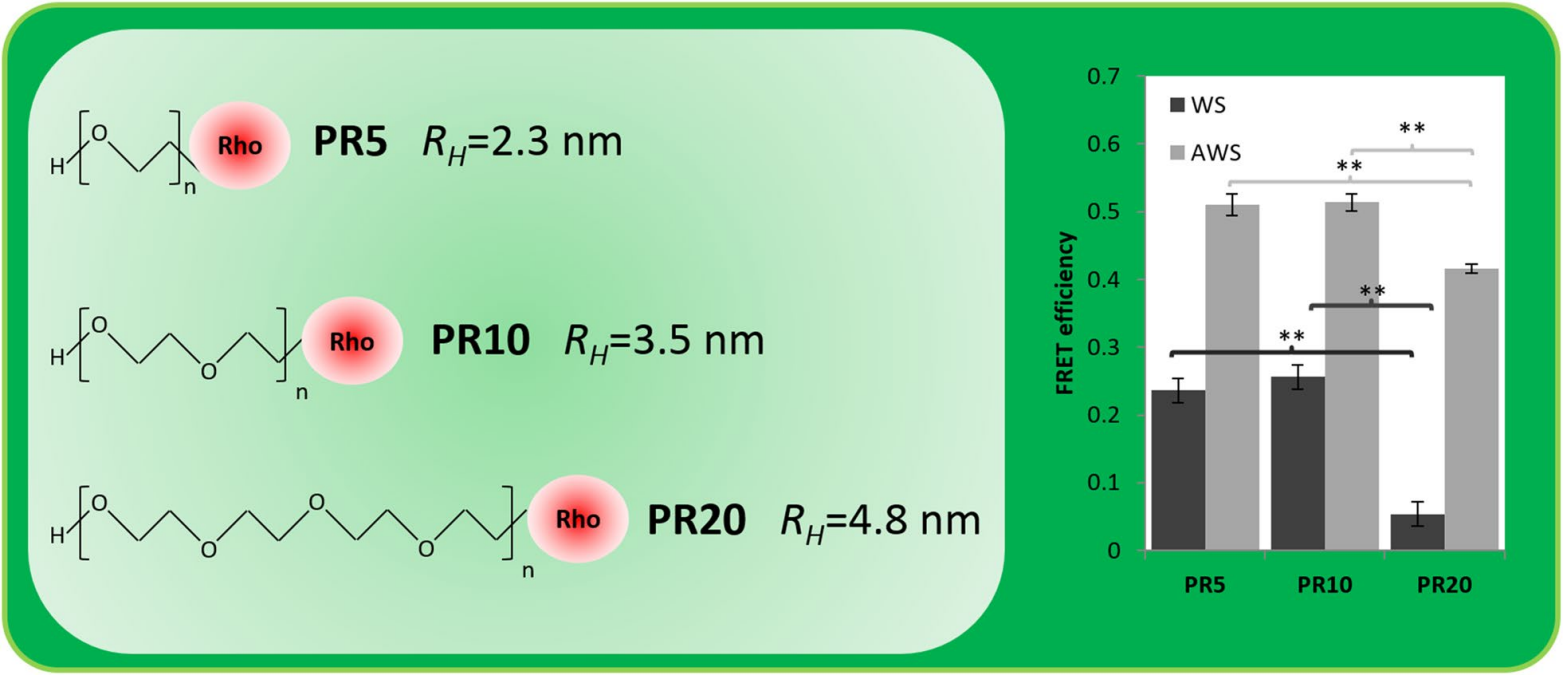
Comparison of the FRET efficiency depending on the PR size and the wheat straw pretreatment. **Mean statistical significant difference with *p *< 0.05

To further demonstrate the amenability of this method to assess enzymes accessibility upon plants chemical treatments, the same methodology was applied to two types of plant samples (Fig. 4): WS and acid-treated WS (AWS), in combination with PR of different molecular weights: 5 kDa, 10 kDa and 20 kDa, named PR5, PR10 and PR20. Their hydrodynamic radius were 2.4, 3.5 and 4.8 nm, respectively. In comparison to WS, AWS composition was modified since hemicelluloses were removed by the acid treatment [39], resulting in better accessibility to cellulose and higher relative concentration of lignin [4]. For WS, comparison of FRET efficiency shows that interactions of PR10 is close to that of PR5, each being five times higher than interaction of PR20. The difference between FRET efficiencies of PR20 and PR5/PR10 are statistical significant (*p *< 0.05). So in the case of an increase in size of the probe above a threshold (around 3.5 nm which is the *R*_H_ of PR10), interactions become much less favourable, which might originate from sterical constraints since such an untreated plant cell wall sample does not show a large porosity.

Interestingly, FRET efficiencies of PR probes with AWS are much higher than with WS: values are 2-times higher for PR5 and PR10 with AWS than with WS and 8 times higher for PR20 (FRET efficiencies of PR20 and PR5/PR10 are also statistically different with *p *< 0.05). The global higher FRET efficiency observed in AWS samples in comparison to WS samples is most likely due to the effect of pretreatment. Removal of hemicelluloses in AWS improves molecular porosity, while lignin is partially unmasked, thus making it more accessible [3]. These results demonstrate that the FRET-SLiM approach is relevant to evaluate the accessibility of lignin by the use of a series of fluorescent probes such as rhodamine-PEGs. Moreover, it demonstrates that interactions of PR can be finely evaluated depending on their size, revealing threshold that are difficult to determine otherwise and might be useful to understand the diffusion and interaction behaviour of enzymes involved in the hydrolysis of lignocellulose.

## Conclusions

Interactions of fluorescent probes with native and pretreated plant cell wall sections were successfully carried out using the SLiM technique, which allows for the first time a quantitative, sensitive and unambiguous determination of molecular interaction. Furthermore, this method is non-destructive and can be applied to dynamic interactions studies. This is an important achievement demonstrating the possibility to use cell wall autofluorescence, despite its spectral complexity, as a FRET donor for acceptors such as rhodamine-based fluorescent probes. Thus, it now might be extended to other biomass species variously pretreated and possibly to other compatible fluorophores. Even more interestingly, interactions of lignocellulose-active enzymes could be assayed in plant cell walls by spectral fluorescence lifetime imaging microscopy, resulting in the possibility to determine enzymes interacting with plant cell wall depending both on their localization at the cellular scale and on their interaction strength. As a consequence, such analysis should pave the way for revealing the impact of pretreatment on enzyme chemical and structural accessibility and for proposing new strategies for enzyme designing aiming at limiting non-specific interactions with plant cell wall.

## Methods

### Sample preparation

PEG-rhodamine of 5, 10 and 20 kDa (PR5, PR10 and PR20) (Creative PEGWorks, USA) and dextran–rhodamine of 10 kDa (DR10) (Sigma-Aldrich, Saint-Quentin Fallavier, France) were selected as the fluorescent probes. Their hydrodynamic radius (*R*_H_) was measured as previously done [19]. Transverse sections of 60 µm-thickness wheat straw sample were cut using a microtome equipped with disposables blades (Microm Microtech HM360, France). Samples were prepared as previously described [39]. Sections were incubated for 72 h at room temperature in 0.01% w/v fluorescent probe in 30 mM phosphate buffer pH 6.0. Sections were mounted between a cover glass and a #1.5H cover-slip.

### Multiphoton confocal microscopy

Spectral images were acquired using laser scanning microscope LSM 710 NLO Zeiss (Zeiss SAS, Germany) coupled with a Chameleon TiSa accordable 80 MHz pulsed laser (COHERENT, USA). Sample excitation was performed at 750 nm with two photon laser and spectral images were acquired using spectral detector (32 channels simultaneously) of the microscope between 420 and 722 nm. Fuorescence images presented in this paper were spectral colour coded representation (each of the 32 channels was represented by his corresponding colour from blue to red).

### Spectral fluorescence lifetime imaging microscopy (SLiM)

Lifetime measurements were acquired using a MW-FLIM detector system along the SPC 150 photocounting card from Becker & Hickl (Becker & Hickl, Berlin, Germany). This system allowed to perform lifetime measurements along 12.5 ns time windows with a 16 spectral channels detector (PML 16) (see Fig. 1) and was driven by SPCM software (Becker & Hickl).

### SLiM calibration

Based on measurements performed on hydroxy-urea crystals, the system exhibits a time response with a FWHM in the order of 170 ps which is perfectly adapted for biological applications [35], especially when using the fitting function of Eq. 1.

The SLiM detection system included a spectral grating which dispatched input photons according to their wavelengths on 16 different spectral channels. The following calibration procedure allowed determining the different spectral windows for each channels. To determine the window spectral width of a channel, we used second harmonic signal generation (SHG) of urea crystal. In fact, SHG signal is emitted exactly at the middle wavelength of used excitation wavelength. By scanning the excitation wavelength from 900 to 980 nm, the first corresponding emission wavelength channels of SLiM could be determined precisely. Then the wavelength width was determined to be equal to 12.5 nm and the wavelength width from channel 3–16 could be determined precisely by iterations.

Confirmation of the spectral channels width was done using mirror sample and the visible continuous lasers of the confocal microscope. Each visible laser rays (458 nm, 488 nm, 514 nm, 561 nm and 633 nm) was perfectly detected by reflection in the expected SLiM spectral channels. For the complete characterization procedure, refer to the following articles [34, 35].

### Lifetime measurements

Samples were excited using 750 nm wavelength and lifetime trace of emitted photons was accumulated during 30 s simultaneously on all spectral channels of the MW-FLIM detector. Each lifetime trace was acquired on 1024 temporal channels. Lifetime traces were then processed using SPCMImage software (Becker & Hickl). By modelling the experimental traces with the following bi-exponential incomplete model (Eq. 1), *a*_1_, *t*_1_, *a*_2_, *t*_2_ parameters were determined. IRF corresponds to the instrumental response function of the FLIM system.

1 $$F\left( t \right) = IRF \otimes\left(a_{1} e^{{ - \frac{t}{{t_{1} }}}} \left( {1 + \frac{1}{{e^{{\frac{12.5}{{t_{1} }}}} - 1}}} \right) + a_{2} e^{{ - \frac{t}{{t_{2} }}}} \left( {1 + \frac{1}{{e^{{\frac{12.5}{{t_{2} }}}} - 1}}} \right)\right)$$

Then the mean lifetime *T*_m_ could be calculated using Eq. 2:

2 $$T_{m} = \frac{{a_{1} t_{1} + a_{2 } t_{2} }}{{a_{1} + a_{2} }}$$

The FRET efficiency E_FRET_ was evaluated using Eq. 3:

3 $$E_{FRET} = 1 - \frac{{T_{mDA} }}{{T_{mD} }}$$

where *T*_mDA_ is the mean lifetime of the donor sample in presence of acceptor and *T*_mD_ is the mean lifetime of the donor considered alone.

### Statistical analysis

Statistical analysis of FRET efficiencies data were performed using Man Whitney test.

## Additional files

**Additional file 1. Figure 1**. Spectral analysis of sample fluorescence. Fluorescence contour maps of (a) WS and (b) rhodamine B; (c) spectral emission of WS alone and in the presence of fluorescent probes.

**Additional file 2. Figure 1**. Detailed SLiM data of WS, WS + PR10, WS + DR10 for the 16 channels analysed.

**Additional file 3. Figure 1**. SLiM analysis of channel 2 indicating lifetime decay for WS, WS + PR10 and WS + DR10.

## Abbreviations

FRET: fluorescence (or Förster) resonance energy transfer
SLiM: spectral and lifetime measurement
PR: PEG-rhodamine
DR: dextran–rhodamine
WS: wheat straw
AWS: acid treated wheat straw
