# Supplementary figures and images for: FRET-SLiM on native autofluorescence: a fast and reliable method to study interactions between fluorescent probes and lignin in plant cell wall
Source: Plant Methods. 2018 Aug 27;14:74. doi: 10.1186/s13007-018-0342-3 (PMC6109981; doi:10.1186/s13007-018-0342-3)

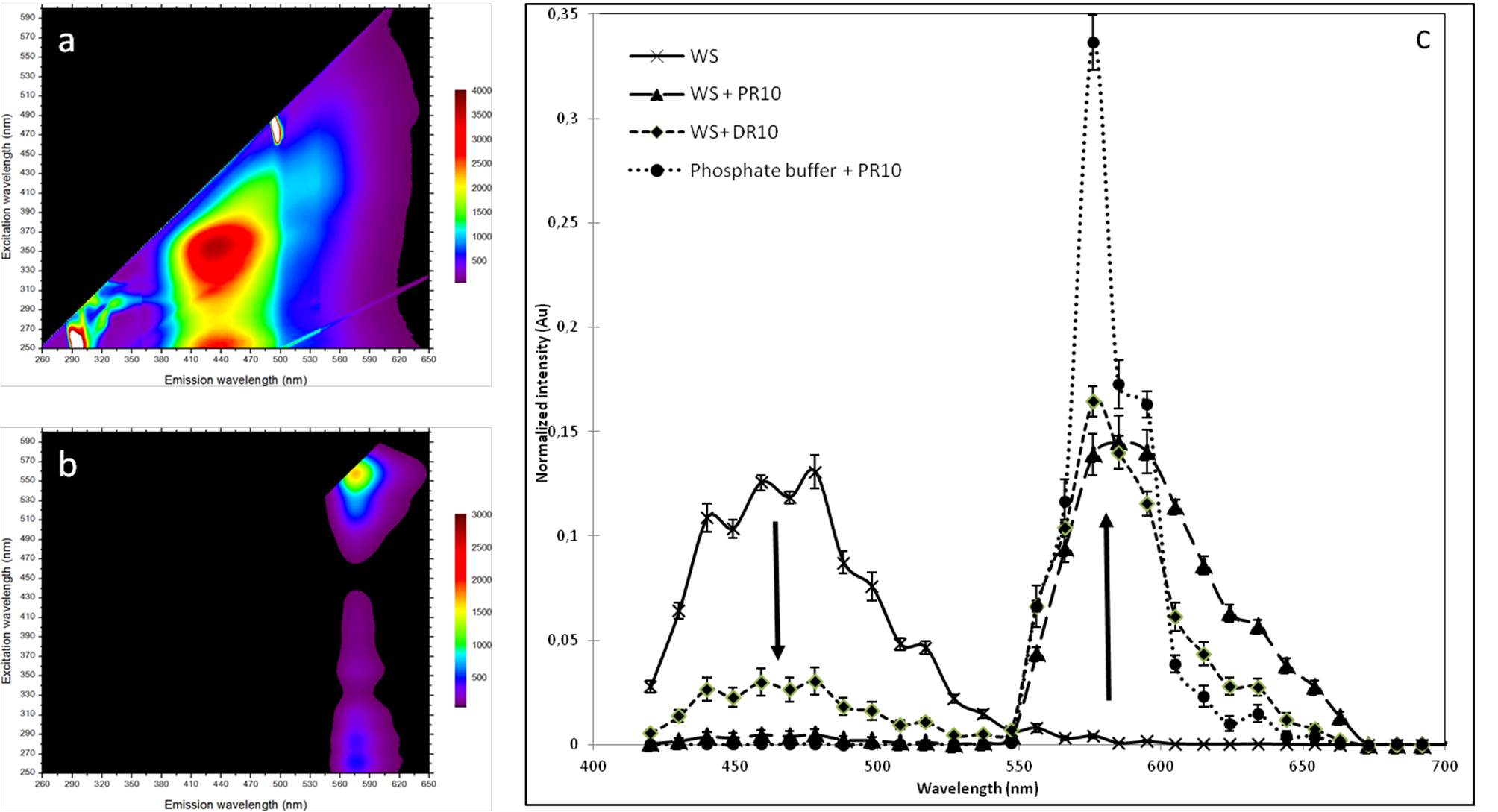

Supplement: Supplementary file 1 — Additional file 1. Figure 1. Spectral analysis of sample fluorescence. Fluorescence contour maps of (a) WS and (b) rhodamine B; (c) spectral emission of WS alone and in the presence of fluorescent probes. [file 13007_2018_342_MOESM1_ESM.tif]

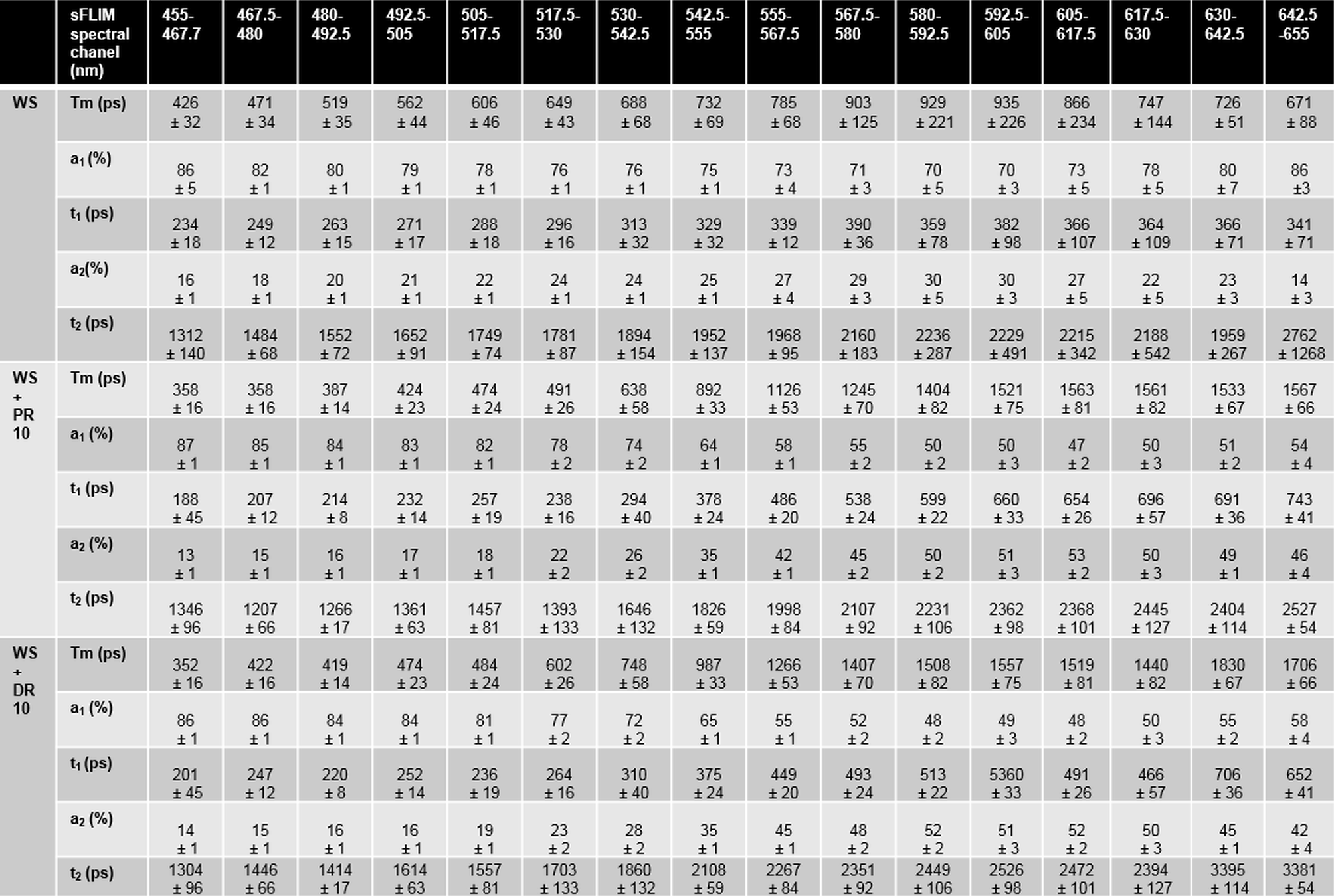

Supplement: Supplementary file 2 — Additional file 2. Figure 1. Detailed SLiM data of WS, WS + PR10, WS + DR10 for the 16 channels analysed. [file 13007_2018_342_MOESM2_ESM.tif]

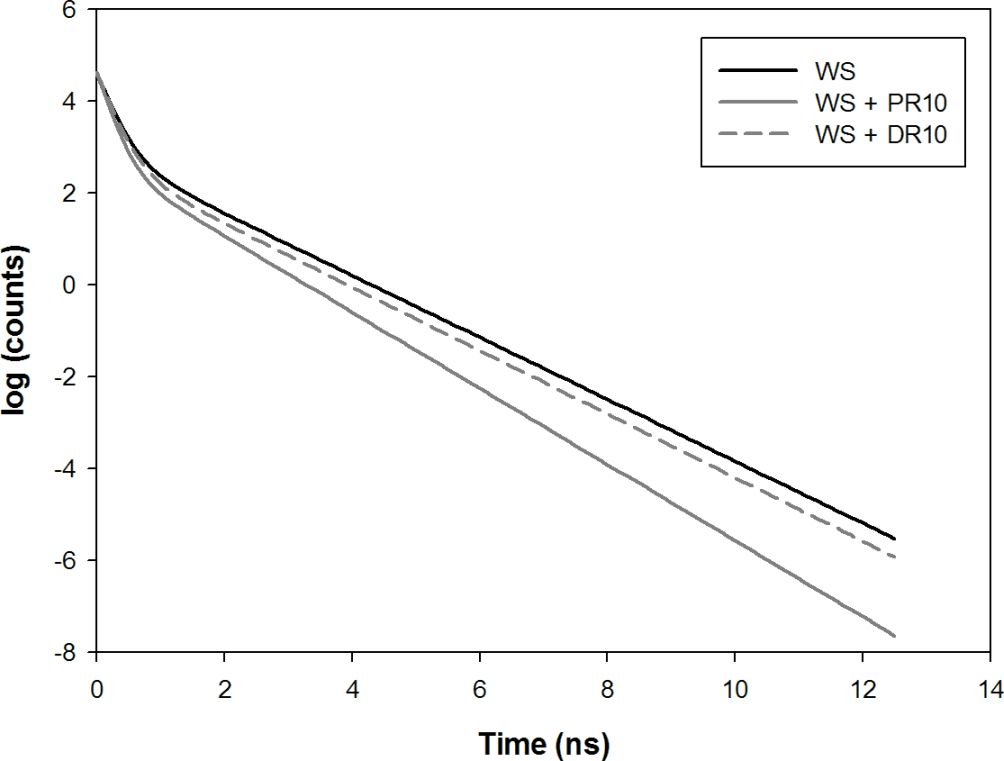

Supplement: Supplementary file 3 — Additional file 3. Figure 1. SLiM analysis of channel 2 indicating lifetime decay for WS, WS + PR10 and WS + DR10. [file 13007_2018_342_MOESM3_ESM.tif]
